# Unsupervised Meta-Analysis on Diverse Gene Expression Datasets Allows Insight into Gene Function and Regulation

**DOI:** 10.4137/bbi.s665

**Published:** 2008-05-26

**Authors:** Julia C. Engelmann, Roland Schwarz, Steffen Blenk, Torben Friedrich, Philipp N. Seibel, Thomas Dandekar, Tobias Müller

**Affiliations:** Department of Bioinformatics, Biocenter, University of Würzburg, Am Hubland, D-97074 Würzburg, Germany

**Keywords:** Arabidopsis thaliana, microarray, unsupervised meta-analysis, function prediction, database, gene expression

## Abstract

Over the past years, microarray databases have increased rapidly in size. While they offer a wealth of data, it remains challenging to integrate data arising from different studies. Here we propose an unsupervised approach of a large-scale meta-analysis on *Arabidopsis thaliana* whole genome expression datasets to gain additional insights into the function and regulation of genes. Applying kernel principal component analysis and hierarchical clustering, we found three major groups of experimental contrasts sharing a common biological trait. Genes associated to two of these clusters are known to play an important role in indole-3-acetic acid (IAA) mediated plant growth and development or pathogen defense. Novel functions could be assigned to genes including a cluster of serine/threonine kinases that carry two uncharacterized domains (DUF26) in their receptor part implicated in host defense. With the approach shown here, hidden interrelations between genes regulated under different conditions can be unraveled.

## Introduction

In the last years, enormous data has been generated with microarray experiments from different organisms, tissues and platforms under various experimental conditions. Databases like the NCBI Gene Expression Omnibus (GEO) (Barrett et al. 2007), ArrayExpress (Parkinson et al. 2007) and NASCArrays (Craigon et al. 2004) have been set up to archive these datasets and to make them available to the scientific community. The size of microarray databases is likely to increase exponentially in the future, as is typical for all molecular databases, increasing the need for sophisticated methods to analyze these large amounts of data appropriately.

Several factors impede a straight-forward analysis of microarray database content: standards for data submission vary between different databases, some microarray datasets do not provide raw data and on the experimental side, protocols and experimental conditions can differ between diverse laboratories conducting microarray hybridizations. However, a major advantage of microarray meta-analysis is that through the integration of a potentially large number of datasets, additional insights into gene regulation can be gained which could have been overseen or not detected in the single experiments. Reasons for this could be that either the signal from a particular gene or group of genes was too weak to be detected in the single experiment or because it can be put into a functional context taking into consideration its regulation under other conditions or treatments.

Several methods for microarray meta-analysis been proposed in recent years, most of them using models which compute an “effect size” and take care of inter-study variation (Choi et al. 2003; Conlon et al. 2006; Hu et al. 2005; Moreau et al. 2003). Thus, they often resemble procedures applied for the detection of differential expression but add the study as an extra explanatory variable. Several datasets from different microarray experiments are integrated in the meta-analysis to increase the number of replicates and thereby the power to detect differentially expressed genes. Because this design implies that datasets addressing the same topic such as the same cell type or treatment are used, microarray meta-analyses of this kind usually consist of only a small number of studies.

A second approach to supervised microarray meta-analysis is to integrate knowledge of biological functions into the analysis to predict global co-expression relationships and to infer functional relationships between co-regulated genes (Huttenhower et al. 2006).

Nevertheless, all the above methods are based on parametric models which have several biological and statistical assumptions. Similar to classical microarray analysis, in which a first explorative analysis reveals possible signals in the data which can then be verified or disproved by parametrical hypothesis testing, our approach of unsupervised meta-analysis yields insights into the biological structure of the data and may thus lead to precise biological hypotheses. These could then be tested by the parametric models described above. The aim of this study is to compare the results from a large number of microarray experiments on *Arabidopsis thaliana* using the well established Affymetrix ATH-1 Genome Array^1^ as a starting point. We restricted our analysis to this highly-standardized platform to reduce uninformative variability introduced by different technologies.

In this unsupervised meta-analysis, we show how to overcome the challenges posed by the heterogeneity of microarray data and apply exploratory data analysis methods. First, microarray datasets from public web sources were collected and pre-processed to remove noise from the data and build a common data basis for further analyses. Later, exploratory data analysis was applied to the processed datasets, namely kernel Principal Component Analysis (kPCA) and spectral and hierarchical clustering, to group contrasts from different microarray experiments and to find genes regulated in a specific cluster. Identification of regulated genes in a specific cluster was achieved by unsupervised feature subset selection using the kernel principal component loadings. Although gene selection or feature subset selection is a challenging task for classification, many different approaches have been proposed for the same. According to our knowledge, gene selection or feature subset selection has not yet been performed using loadings of features on kernel PCA scores in the context of meta-analysis.

Genes selected to play a role in either plant growth and development (related to indole-3-acetic acid, a plant growth hormone) or pathogen defense were mapped onto physiological processes and functions and could be validated by previous studies. For genes which have not completely been characterized yet, our approach was able to propose a function and a possible regulatory mechanism as shown here for DUF26 (Domain of Unknown Function) kinase genes.

## Methods

### Data pre-processing

**Figure 1:**
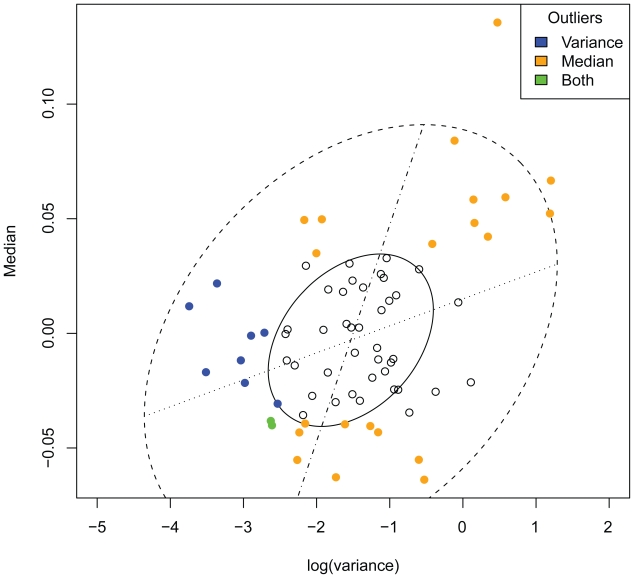
**Outlier removal.** Median vs. log (variance) plot of all 76 contrasts and the associated bivariate box plot, colors indicate the type of outlier (see legend). The bivariate box plot is the two-dimensional analog of the familiar box plot of univariate data and consists of a pair of concentric ellipses, the hinge and the fence (Everitt 2005). This box plot is based upon a robust estimator for location, scale and correlation. Uncolored contrasts were kept for further analysis.

Microarray data were collected from the Gene Expression Omnibus (GEO) database (Barrett et al. 2007). For our analysis, we defined a *dataset* as a GEO entry with a unique GSE series accession number. Each dataset consisted of several Affymetrix CEL-files, each one representing the raw data from one microarray hybridization. The raw data of one microarray is termed a *sample* in the following section. Instead of comparing whole GEO datasets with each other, we broke down each dataset into *contrasts* and used these as ‘entities’ for our analysis (Fig. 1). A *contrast* is the difference in gene expression between any two sample groups of the same dataset. A sample group contains all replicate samples from one condition (e.g. treatment, mutant, see Table 1). Therefore, for most GEO datasets, several contrasts were set up. For example, a contrast could be a comparison of an *Arabidopsis thaliana* mutant with a wild type plant.

A contrast was then represented by a vector of the logarithmic (base 2) fold changes of all 22810 probe sets on the ATH1 chip. The majority of probe sets on the ATH1 chip interrogates the expression level of one gene, some match to two or more genes. Before computing the fold changes, raw intensity values of all samples of a contrast were normalized using the gcRMA algorithm implemented in the *gcrma* package (Wu et al. 2005) which is part of Bioconductor (Gentleman et al. 2004) and runs under the statistical software R (R Development Core Team 2004). Logarithmic fold changes and p-values adjusted for multiple testing using the false discovery rate method (Benjamini and Hochberg 2000) were computed using the *limma* package (Smyth 2004) which is also integrated into Bioconductor.

We imposed the following selection criteria on the datasets: a) Availability of the Affymetrix raw data (CEL-files) for download, b) at least two replicates of each condition are available c) time-course experiments were excluded. 20 GEO datasets fulfilled these criteria as of November 2006. From these datasets, 76 contrasts could be set up on the basis of 424 CEL-files. The final data matrix used for the unsupervised meta-analysis was a 76 × 22810 matrix, 76 contrasts with 22810 log fold changes.

### Outlier removal and transformation

To remove experimental outliers from the data which could negatively influence any further analysis, a filtering criterion was set up as follows. Across all experiments, 15% and 85% quantiles of the distributions of medians and variances of the log fold changes were calculated. Experiments whose medians laid outside the inter-quantile-range or whose variances were below the 15% quantile threshold were excluded from further analysis. This resulted in a reduced data matrix *X* with 41 remaining contrasts. We randomly inspected the 35 removed contrasts for detectable problems and found several contrasts having a low-variant distribution of multiple-testing corrected p-values with almost all p-values close to one.

When dealing with heterogenous experimental datasets from different laboratories and experimental settings, efficient data transformation methods are necessary to produce a reasonable level of comparability. Log fold changes from microarray experiments deserve special attention in that they implicitly define a “direction” of differential expression by their algebraic sign which is semantically not sustainable when comparing contrasts from divergent settings. We therefore only evaluated the absolute value of the log fold changes and brought all remaining 41 contrasts approximately to a standard normal distribution by applying the *Box-Cox-Transformation* (Eq.1, (Box and Cox, 1964)) using Maximum-Likelihood estimated power coefficients.

For a power coefficient *p* and data *x* the box-cox-transformed data *x*′ is defined as follows:

(1) $$x^{\prime }=\{\begin{array}{ll} (x^{p}-1)/p & if\;p\neq 0\\ log(x) & if\;p=0 \end{array}$$

The average *p* values were about 0.13, resulting in an approximately logarithmic transformation of the log fold changes. Subsequently, all datasets were standardized to zero mean and unit variance to analyze datasets without regard to their scale and location.

### Kernel PCA

Principal Component Analysis (PCA) aims to provide a lower dimensional view of high dimensional data by projecting the data points from a data matrix *X* onto a new co-ordinate system retrieved by eigen-decomposition of the associated covariance matrix. The axes of the new coordinate system are thereby chosen in a way that each axis or principal component explains as much of the (remaining) variance of the data as possible and that all axes after the first are orthogonal to the ones before.

Kernel PCA (kPCA) (Schölkopf et al. 1998) is a non-linear extension of the regular PCA, performing the same projection in a possibly even higher dimensional feature space. The data points are implicitly projected from the input space *I* into the feature space *F* by replacing the standard Euclidean dot product with a positive-semidefinite symmetric bilinear form, the kernel function *κ* (Eq. 2). The algorithm is represented in a dual form such that all computation takes place using only the matrix of pairwise dot products *XX*′ (Shawe-Taylor and Cristianini 2004), the Gram or Kernel matrix *K* (Eq. 3), instead of using the data points or its variances directly.

More precisely, for a row-indexed data matrix *X* and a mapping *φ: I* → *F, x* ↦ *φ*(*x*) the kernel function *κ* and its associated kernel matrix *K* is defined as

(2) $$\kappa (x_{i},x_{j})=\langle \varphi (x_{i}),\varphi (x_{j})\rangle$$

(3) $$K_{ij}=\kappa (x_{i},x_{j}).$$

kPCA has the advantage of being able to detect non-linear patterns in the data which might be overlooked or not covered appropriately when using conventional PCA.

For our analysis we used the kPCA algorithm implemented in the “kernlab” package (Karatzoglou et al. 2004), for the kernel function κ we chose a polynomial kernel

$$\kappa (x_{i},x_{j})={\left(s\langle x_{i},x_{j}\rangle +k\right)}^{d}$$

of degree *d* = 2, scale *s* = 1 and offset *k* = 0.

### Clustering

Clustering was performed on all remaining contrasts after removal of outliers. For an initial identification of the three main clusters of contrasts, we applied a spectral clustering algorithm from the “kernlab” package (Karatzoglou et al. 2004). Spectral clustering algorithms cluster points using eigenvectors of matrices derived from the data, the kernel matrix *K* in this case. Similar to k-means clustering for data in the input space, the initial number of clusters has to be specified.

To gain structured clustering results, we applied hierarchical clustering using Ward’s minimum variance method, which aims to find compact and spherical clusters based on Euclidean distance (Ward 1963). Decomposition of the symmetric kernel matrix *K*

(4) $$K=S\Lambda S^{\prime }$$

leads to a product of the orthogonal matrix *S* of its eigenvectors, a diagonal matrix Λ consisting of its eigenvalues and the transpose of *S*, *S*′. As the eigenvalues of *K* are directly linked to the proportion of explained variance of the principal component axes, the axes were scaled by the square roots of their respective eigenvalues, i.e.

(5) $$\tilde{X}=S\Lambda^{1/2}.$$

The result is a Euclidean distance

(6) $$d(x_{i},x_{j})=\sqrt{\langle {\tilde{x}}_{i},{\tilde{x}}_{j}\rangle }$$

weighted by the information content of each of the vector coefficients, thus scaling down axes that were given a low information content in the previous kPCA analysis.

Uncertainty of the predicted clusters was estimated by a 1000-fold multi-scale boot-strap resampling using the “pvclust” algorithm (Suzuki and Shimodaira 2006).

## Results

### Dimension reduction by kernel principal component analysis (kPCA)

The ATH-1 whole genome chip consists of 22810 probe sets, this led to a 41 × 22810 data matrix (contrasts × log fold changes of probe sets) after outlier removal. To reduce the dimension of the data matrix, a kPCA algorithm was applied which was able to cover virtually the complete information content by defining an orthonormal system of 38 principal component axes. The 22810 log fold changes could therefore be represented by a 41 × 38 data matrix without any measurable loss of information. Using only the first 25 principal components, 80.585% of the variance could be described. If we state that the remaining 20% of the variance in the data describe noise, an estimation which is certainly not too strict in the context of large-scale gene expression measurements, an effective de-noising can be reached by considering only the first 25 principal components in further steps of the analysis. For a detailed overview of the variance distribution on the first 15 principal components, see Table 2.

### Unsupervised analysis reveals three clear clusters of contrasts

**Figure 2:**
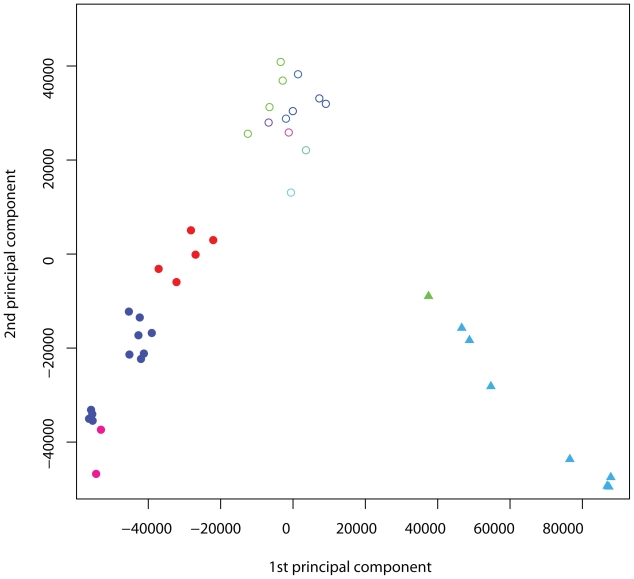
**Kernel PCA on 41** ***Arabidopsis thaliana*** **contrasts.** Plot of all 41 contrasts using the first two principal component axes. Comparisons are colored according to the experiment they originated from and correspond to the colors used in Figure 3, different shapes indicate the three different clusters obtained from spectral clustering: Indole-3-acetic acid (IAA) related contrasts (solid circle), pathogen related contrasts (triangles) and others (outlined circle).

The principal component plot (Fig. 2) revealed three major clusters of contrasts and several minor ones. In contrast to typical meta-analyses these clusters were not a priori defined, but detected by the proposed unsupervised meta-analysis. Based on this clustering we used an implementation (Karatzoglou et al. 2004) of the spectral clustering algorithm proposed by Ng et al. (2001), a variant of the k-means clustering algorithm in a kernel defined feature space, to support the clusters shown in Figure 2. According to the annotation of the datasets retrieved from GEO, the three clusters were related to indole-3-acetic acid (IAA) addition or inhibition (cluster 1, triangles), pathogen defense activation (cluster 2, solid circles) and “others” (cluster 3, outlined circles). For a detailed biological interpretation, see section “Biological interpretation of clusters”. Additionally, inspection of the pairwise plots of the other principal components contributing to a lower extent to the variance of the data revealed more contrast clusters.

**Figure 3:**
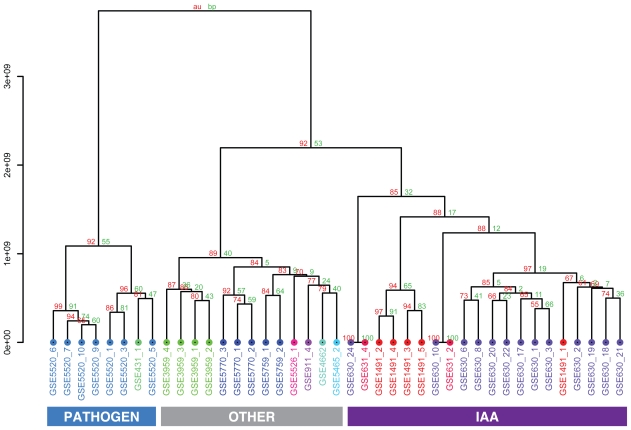
**Hierarchical clustering on 41** ***Arabidopsis thaliana*** **contrasts.** Cluster dendrogram using hierarchical ward clustering on all 38 principal component vectors resulting from kernel PCA. Contrasts are colored according to their experimental affiliation. Approximately unbiased (au), (Suzuki and Shimodaira 2006) and standard bootstrap (bp) values are given for all splits and support the results from the previous spectral clustering (Fig. 2).

To get further structural insights into the relationships between contrasts and the experimental settings, we performed hierarchical clustering assessed by multi-scale boot-strapping (Fig. 3). In agreement with the spectral clustering performed earlier and the graphical inspection of the pairwise scatterplots of contrasts on the kPCA axes, the three main clusters of contrasts could also be found as the first two splits in the resulting dendrogram with high bootstrap support.

As the three clusters were mainly separable through the x-axis on the kPCA scatter-plot using the first two axes (Fig. 2), we postulated that the first principal component alone might be enough to select genes whose co-regulation patterns could clearly distinguish between IAA related, pathogen-defense related and other contrasts.

### Gene selection with kPCA loadings

To accomplish an efficient feature subset selection, i.e. to identify genes that are responsible for the clustering, a variety of methods have been described, e.g. Self-Organizing Maps (SOMs) (Tamayo et al. 1999), Maximal Margin Linear Programming (MAMA) (Antonov et al. 2004), Correlation Based Feature Selection (CFS) (Hall 1999) or Recursive Feature Elimination (RFE) using Support Vector Machines (SVM) (Guyon et al. 2002; Zhang et al. 2006). In consequent continuation of our approach of exploratory meta-analysis, we looked for genes that have a strong association with the first kPCA axis, i.e. we calculated the loadings of each of the genes onto the principal components. To achieve this with respect to the kernel defined feature space we projected single artificial contrasts containing only one de-regulated gene onto the new coordinate system. Each of the 22810 artificial contrasts was set up in a way that it showed a high absolute fold change value in one of the genes and all others being set to zero. From the resulting 22810 × 38 matrix of loadings of each of the genes onto the 38 principal components, we selected the 500 top genes for both positive (IAA related) and negative (pathogen related) extrema. To assess the accuracy of the gene selection process exploratively, we repeated the previous kPCA analysis using only the selected genes, i.e. on the remaining 41 × 500 data matrices, and inspected pairwise scatterplots of the first 20 principal components for each data-set of either IAA-related or pathogen-associated genes. All kPCA plots of the IAA-related gene set, even the one of the first two axes which contribute most to the overall variance of the data, showed a wide spread of IAA contrasts along the principal component axes. This indicated a high variance of the selected genes in IAA-related contrasts. All other contrasts were projected onto a compact local cluster by kPCA, demonstrating that the selected genes do not vary in these contrasts. The same was found in the kPCA plots of the matrix with pathogen-associated genes (data not shown). These findings indicate that expression patterns related neither to IAA nor pathogen treatment were efficiently stripped off by the gene selection process.

### Biological interpretation of clusters

The hierarchical clustering on all kPCA scores in Figure 3 revealed three main clusters of contrasts: contrasts studying pathogen defense (blue), contrasts analyzing indole-3-acetic acid (IAA) effects (violet) and other contrasts studying various effects (gray). These three clusters were well-supported by high bootstrap values. The labels at the edges include the GEO accession number followed by an index indicating the contrast number. For a detailed description of contrasts see Table 1. For each contrast, two groups of samples were compared and for each group, the genetic background and treatment is listed. The last column of Table 1 indicates the cluster this contrast was assigned to in kPCA clustering.

Zooming into the IAA cluster, a cluster containing only contrasts with IAA inhibition (GSE1491_2, GSE1491_3, GSE1491_4 and GSE1491_5) was well-separated from the remaining contrasts, including GSE1491_1, a contrast from the same dataset, but where IAA instead of an IAA inhibitor was added to one sample group. The remaining contrasts in the IAA cluster mainly studied the effect of IAA on different mutants with defects in IAA biosynthesis or signaling. Indole-3-acetic acid (IAA) belongs to a group of plant growth hormones called auxins. The “others” – cluster consisted of contrasts studying various effects like the effect of lincomycin which is an inhibitor of plastid protein translation, regulation changes of an embryogenesis transcription factor mutant or of stress tolerant mutants. Naturally, in this cluster of divergent contrasts, contrasts from the same dataset clustered closely together. The architecture of the hierarchical cluster tree shows that data preprocessing followed by kPCA adjusted the data in such a way that contrasts stemming from biologically similar experiments are indeed more similar to each other than to other contrasts. Thus, with our analysis, we were able to achieve comparability of microarray datasets from different laboratories addressing different biological questions. This is nontrivial and important considering the numerous sources of variation that affect the nature of the datasets underlying this analysis.

#### *Arabidopsis thaliana* genes regulated by indole-3-acetic acid (IAA)

To get an overview of the functions of the selected genes representative for the contrast clusters “IAA” or “pathogen”, the *Arabidopsis thaliana* pathway analysis program MapMan (Usadel et al. 2005) was used. With MapMan, gene expression values can be displayed onto diagrams of functional categories and metabolic and regulatory pathways. In this study, MapMan was used to visualize the representative genes for the two clusters “IAA” and “pathogen”.

Among the genes representative for IAA contrasts, the functional category “hormones” with the subgroup “IAA” defined by MapMan showed the highest proportion of regulated genes (diagram not shown). The subgroup “IAA” consists of 215 genes in MapMan. We selected 500 genes representative for IAA with our approach and out of these, 43 genes are cataloged in the MapMan subgroup “IAA”. Thus, by selecting 500 genes from the ATH1 microarray which comprises roughly 2% of the array, we were able to capture 20% of the genes annotated as IAA-related in MapMan.

In the “hormones” subgroup “ethylene”, and in the category “transcription factor” many genes are regulated under IAA treatment, while a smaller number of genes is regulated in the categories “Cytochrome P450” and “cell wall” (data not shown).

Regulated genes in the subgroup “ethylene” are either involved in ethylene synthesis or signal transduction. Ethylene plays a role in the regulation of a number of developmental processes, often in interaction with other plant hormone signals. For example, auxins can induce ethylene formation and in turn ethylene can trigger an auxin increase. Some processes such as root elongation, differential growth in the hypocotyl and root hair formation and elongation are regulated by both auxin and ethylene in *Arabidopsis thaliana* (Stepanova et al. 2005). All the GEO datasets we annotated as IAA-related originate from seedling RNA extracts. Since IAA belongs to the group of auxins, the aforementioned processes are likely to be regulated under IAA treatment.

Cytochrome P450 monooxygenases are involved in various biosynthetic reactions which synthesize for example plant hormones or defense compounds. Regulation of cell wall genes is also expected as auxins mediate cell elongation by stretching of the cell wall which requires restructuring processes.

In conclusion, the gene selection of our unsupervised meta-analysis approach chose many genes which are annotated and independently validated as being IAA regulated.

#### *Arabidopsis thaliana* genes regulated by pathogen exposure

**Figure 4:**
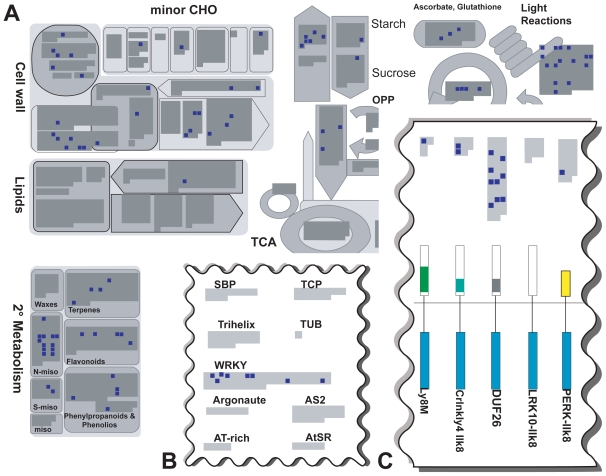
**Overview of genes regulated in pathogen associated contrasts.** The gray areas inside the individual diagrams of the functional categories represent all genes present on the ATH1 chip. Dark blue squares highlight genes regulated in contrasts of the “pathogen” cluster. **A)** Regulation of cell wall genes (upper left), alkaloids which fall into the category “N-misc.” of “secondary metabolism” and “Light Reactions” of photosynthesis (upper right) is apparent. **B)** Part of the “transcription” map indicating regulation of WRKY transcription factors. **C)** Section of the “receptor like kinases” map indicating regulation of DUF26 kinases. Figure reading example: In subfigure C, a total of 41 DUF26 kinases are represented on the ATH1 chip of which 9 are regulated after pathogen exposure. The figure is based on maps from the pathway analysis program MapMan (Usadel et al. 2005).

**Figure 5:**
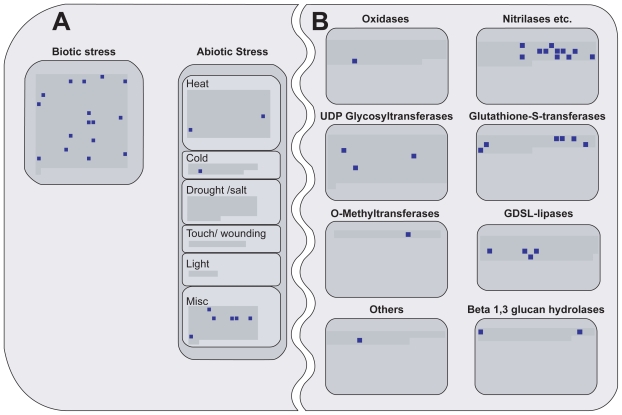
**Overview of (A) stress genes and (B) genes of large enzyme families regulated in pathogen-associated contrasts.** The gray areas inside the individual diagrams of the functional categories represent all genes present on the ATH1 chip. Dark blue squares indicate regulated genes. Subcategories “Biotic Stress” (A) and “Nitrilases etc.” (B) contain a high number of genes regulated after pathogen exposure. The figure is based on maps from the pathway analysis program MapMan (Usadel et al. 2005).

Gene selection for contrasts studying plant response to pathogens revealed a high number of regulated genes in the following functional categories of MapMan (Usadel et al. 2005): “biotic stress”, “receptor kinases”, “photosynthesis” (light reactions), “alkaloid-like proteins” from “secondary metabolism”, “nitrilases”, “cell wall” genes and “WRKY transcription factors”. For all of the functional categories mentioned above, it has been reported that genes in these categories are regulated after pathogen attack and play a role in plant defense. Figures 4 and 5 show details of the MapMan maps which harbor these categories. In the figures, gray areas inside the diagrams represent all the individual genes present on the ATH1 chip and annotated in MapMan. The selected genes representative for contrasts studying the effects of pathogen exposure are highlighted by small dark blue squares. For example, Figure 4C shows that there are 41 DUF26 receptor kinases present on the ATH1 chip, of which 9 are regulated after pathogen exposure. In the following, we give a short description of the functions of the genes regulated after pathogen exposure.

A change in carbohydrate metabolism after pathogen attack as observed here (Fig. 4A, upper right: “light reactions”) has also been reported by Berger et al. (2004) for the pathogens *Pseudomonas syringae* and *Botrytis cinerea*. The authors have shown a co-regulation of defense, sink and photosynthetic gene expression in response to the pathogens under study.

As the cell wall is a natural barrier for plant pathogens, plant defense includes cell wall modifications and biosynthesis to thicken cell walls and impede further pathogen attack (Cheong et al. 2002). Figure 4A shows that several genes of the cell wall metabolism are regulated after pathogen exposure.

The regulation of WRKY transcription factors (Fig. 4B, upper left) is also described in the publication accompanying the GEO dataset GSE5520 (Thilmony et al. 2006). Our findings confirm their suggestion that these transcription factors regulate plant response to bacteria.

Alkaloids (Fig. 4A, lower left) are secondary metabolites listed in the “N-misc.” category of MapMan. They are generally not essential for the basic metabolic processes of the plant but play an important role in plant defense (Dixon 2001). They are produced by the plant to restrict pathogen feeding. The accumulation of antimicrobial substances is often regulated by signal-transduction pathways which require the perception of the pathogen by a plant receptor encoded by host resistance genes (Dangl and Jones 2001; Piroux et al. 2007). Thus, the regulation of DUF26 containing genes postulated by our analysis of the *Arabidopsis thaliana* transcriptome (Fig. 4C) might reflect their function in pathogen recognition. Receptor kinases are discussed in more detail in the next section.

The functional category “biotic stress” (Fig. 5A) comprises a number of different genes which are annotated to be pathogen related.

Nitrilases (Fig. 5B, upper right) are involved in IAA biosynthesis and catalyze the conversion of indole-3-acetonitrile to IAA. The induction of four *Arabidopsis thaliana* nitrilases by the pathogen *Pseudomonas syringae* has been shown by Bartel and Fink (1994).

Thus, gene selection by unsupervised meta-analysis was able to pinpoint biologically important genes of which many are experimentally validated to be regulated by pathogen attack. Clearly, one could postulate that the remaining genes of unknown function are also associated with responses to pathogen attack.

### Serine-threonine kinases involved in plant response to pathogens

As presented in Figure 4C, the extracted set of genes deregulated in response to pathogens includes a number of receptor kinases. Many kinases belong to the group of serine/threonine kinases of the DUF26 subfamily. They all share the same domain composition and order consisting of a signal peptide, an extracellular region containing two domains of unknown function (DUF26, PF01657) and a cytosolic serine/threonine kinase domain (pkinase, PF00069). According to the SMART database (Letunic et al. 2006), proteins of this family are exclusively found in Streptophyta. The 9 putative receptor kinases exhibit high similarity in domain composition and nucleotide sequence with the receptor-like kinase 4 of *Arabidopsis thaliana* (Swiss-Prot-ID Q9C5T0). This enzyme is reported to be a member of the systemic acquired resistance pathway in higher plants. Its expression can be activated by a regulatory protein induced via pathogen and salicylic acid interaction (Du and Chen 2000). Salicylic acid is a signaling molecule which induces systemic acquired resistance in the host plant (Ryals et al. 1996). These findings suggest a function for the putative receptor-like kinases in host defense processes.

Two of the DUF26 kinase genes (At4g21400, At4g21410) were also regulated in the contrasts from dataset GSE3959 and in one contrast from the dataset GSE5770. In the former dataset, the function of B3 domain protein LEAFY COTYLEDON2 (LEC2) was studied. This transcription factor is required for several aspects of embryogenesis including the maturation phase. In the latter contrast, *abi4* mutant plants were treated with lincomycin and compared to untreated mutants. ABI4 is a transcription factor, lincomycin inhibits plastid protein translation. From this finding it may be concluded that these two DUF26 kinase genes either play a role in more than one signaling pathway or that the same pathway is used to regulate several functions. This might be an interesting starting point to study these pathways in more detail.

**Figure 6:**
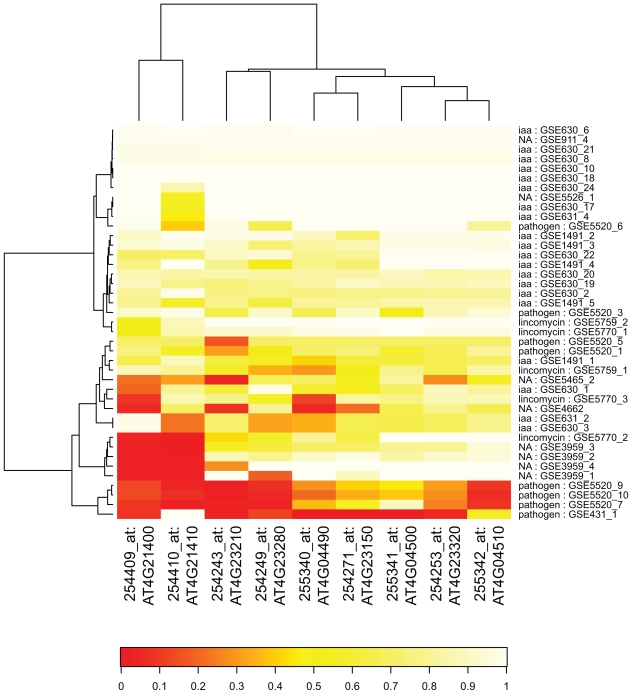
**Regulation of DUF26 kinase genes.** Red cells indicate low p-values for a gene in a particular contrast, light yellow cells represent high p-values. The DUF26 kinase genes are strongly regulated in four pathogen-associated contrasts.

As can be seen from Figure 6, the DUF26 kinase genes were not regulated in all of the contrasts involving pathogen exposure. This could be due to several reasons. For example either the variance in the single microarray intensities was so high that differential expression could not be detected in the contrast or the difference in expression levels (i.e. the logarithmic fold change) was too low to be significant because of biological reasons. Again, this finding might be an interesting starting point to analyze the function and regulation of the DUF26 kinase genes.

## Discussion

Public microarray data repositories accumulate large amounts of data which have so far rarely been used for large-scale analyses. Using this wealth of information, additional implications for the function and regulation of genes can be made which could not be derived from single microarray datasets. This stresses the importance of meta-analyses and their benefit over classical microarray experiments.

In this study, we apply a novel approach of an unsupervised meta-analysis on a large number of gene expression microarrays. Before conducting the analysis, we performed a pre-processing which included a conservative outlier removal. kPCA followed by hierarchical clustering, revealed robust and significant clusters of contrasts which reflect similar experimental conditions. Thus we were able to detect biologically important known and unknown factors (e.g. IAA-or pathogen-associated) through an unsupervised analysis.

To find genes specifically regulated in these clusters, a novel approach of gene selection was conceived. Gene selection was performed using loadings of features on kPCA scores, which has to our knowledge not been performed in the context of meta-analysis before. Gene selection based on loadings of features on kPCA scores circumvents a major drawback of most proposed methods of feature selection: They tend to find linear combinations of features, i.e. genes, that separate the given experimental classes best (e.g. different cancer types, etc.). This is challenging as the search space for all possible linear combinations is too large to be searched exhaustively and sophisticated heuristics and optimization methods have to be chosen which likely yield differing results, see e.g. Zhang et al. (2006). An unsupervised analysis as proposed here circumvents this problem efficiently by working directly on the loadings from the kPCA analysis. Eigen-decomposition of the kernel matrix is deterministic and so are the results from our gene selection process, provided the projection is capable of clustering the contrasts appropriately. The genes selected by our feature extraction were found to be representative of a group of contrasts and could in part be experimentally validated. Furthermore, adding random noise to the data did not change the set of selected genes, proving the robustness of the proposed gene selection method.

It is the gene-selection in the first place that benefits most from an analysis across several datasets. Weak regulation signals can easily be overlooked in a single dataset, i.e. the genes will likely receive an insignificant p-value due to their low fold changes compared to a relatively high variance. The situation becomes even worse after a correction for multiple testing has raised the overall p-value level, efficiently removing those subtle signals. In a meta-analysis approach which integrates many datasets, even a small signal that is consistent across several contrasts can be detected. To ensure this surplus and to prevent early losses of information, we used fold changes and not p-values for our analysis. We performed the unsupervised meta-analysis on absolute fold changes to reduce variation introduced by different experimental settings. For example, when there are contrasts in the dataset which compare a surplus of a factor with a control and other contrasts comparing a lack of a factor with another control, we might expect fold changes with opposite signs but still want the contrasts to cluster closely together because the same factor was studied in both. In some cases the direction of the experimental setup was not even apparent from the description of the dataset.

To ensure that results of similar quality could not be obtained by a simpler model and thus to prevent overfitting of the data we compared the results to the ones obtained from traditional linear PCA. Even though linear PCA was also able to detect some of the major clusters in principle, its accuracy as assessed by hierarchical clustering as well as by the gene selection process fell far short of the results from the kernelized version. Additionally, it should be noted that kPCA outperforms the traditional approach significantly, considering that the dimension of the kernel matrix as a matrix of pairwise scalar products between the data points is independent of the dimension of the data, which is 22810 (the number of probe sets) in the case of the ATH-1 arrays.

For a large *Arabidopsis thaliana* microarray dataset, we demonstrate here that gene selection, based on the study of principal components, proposed genes typical for either IAA-or pathogen-associated contrasts. These genes were proved to be related to either IAA effects or plant reactions in response to pathogen exposure by previous studies. Furthermore, starting from our finding that DUF26 kinases are regulated in pathogen-associated contrasts, we applied homology modeling to propose that DUF26 kinases have a function in plant pathogen defense. Further experiments are needed to confirm this hypothesis. Nonetheless, this example demonstrates how unsupervised analysis can aid and guide the next steps of such an analysis.

In general, unsupervised meta-analysis embracing several highly divergent experimental settings can suggest novel gene functions by revealing the regulation of a gene under different conditions. It is noteworthy that these analyses are not restricted to datasets addressing the same topic, but that they profit from the divergence of the experimental settings.

However, it has to be mentioned that an unsupervised meta-analysis is suggestive rather than definitive. But since it is common in classical statistics to precede a supervised, parametric analysis with an explorative approach to check the integrity and quality of the data, we recommend the same here for microarray meta-analyses. Hypotheses from unsupervised analyses can then be tested with supervised methods and biological experiments.

We have shown here that it is feasible to integrate various datasets spanning a large range of experimental questions and originating from various laboratories into a coherent unsupervised analysis. This analysis can be applied to find genes representative of a cluster of related contrasts. Based on expression changes between clusters, the function and regulation of genes can be predicted. Our study is based on the Affymetrix ATH1 Genome Array platform here, but our approach can be transferred to any platform, organisms and experimental design which allows one to compute a logarithmic fold change, e.g. human or mouse microarray datasets. To achieve easy access to our unsupervised meta-analysis results, we intend to set up a database web server where new datasets can easily be added and compared to our curated database of *Arabidopsis thaliana* ATH-1 microarrays.

## Figures and Tables

**Table 1:** Variance of kernel principal components.

	PC1	PC2	PC3	PC4	PC5
*PV*	0.10035	0.05383	0.05003	0.04640	0.03887
*CP*	0.10035	0.15418	0.20422	0.25062	0.28949
	**PC6**	**PC7**	**PC8**	**PC9**	**PC10**
*PV*	0.03725	0.03250	0.03226	0.03142	0.02973
*CP*	0.32674	0.35925	0.39151	0.42293	0.45267
	**PC11**	**PC12**	**PC13**	**PC14**	**PC15**
*PV*	0.02793	0.02699	0.02647	0.02606	0.02470
*CP*	0.48061	0.50761	0.53409	0.56016	0.58486

Variance of the first 15 principal components on the 41 × 22810 data matrix of *Arabidopsis thaliana* microarray data, explaining close to 60% of the variance of the data.

**Abbreviations:** PV: Proportion of Variance; CP: Cumulative Proportion of variance.

**Table 2:** Overview of all contrasts included in the explorative meta-analysis.

Contrast	Sample group 1		Sample group 2		Cluster
	Genetic background	Treatment	Genetic background	Treatment	
GSE1491_1	WT Col-0	IAA	WT Col-0	non	IAA
GSE1491_2	WT Col-0	IAA inhibitor A	WT Col-0	non	IAA
GSE1491_3	WT Col-0	IAA inhibitor B	WT Col-0	non	IAA
GSE1491_4	WT Col-0	IAA/IAA inhibitor A	WT Col-0	non	IAA
GSE1491_5	WT Col-0	IAA/IAA inhibitor B	WT Col-0	non	IAA
GSE3959_1	MU LEC2GR	1h LEC2 induction	MU LEC2GR	non	other
GSE3959_2	MU LEC2GR	4h LEC2 induction	MU LEC2GR	non	other
GSE3959_3	MU LEC2GR	1h LEC2 induction	WT WS-0	4h LEC2 induction	other
GSE3959_4	MU LEC2GR	4h LEC2 induction	WT WS-0	4h LEC2 induction	other
GSE431_1	pmr4-1 MU	non	pmr4-1 MU	powdery mildew	pathogen
GSE4662_1	MU STA1	non	WT	NA	other
GSE5465_2	MU OETOP6B	non	WT	NA	other
GSE5520_1	WT Col-0	DC1318 Cor 10e6	MU STA1	non	pathogen
GSE5520_10	WT Col-0	EcTUV86-2 fliC 10e8	WT Col-0	non	pathogen
GSE5520_3	WT Col-0	DC3000 10e6	WT Col-0	non	pathogen
GSE5520_5	WT Col-0	DC1318 Cor 5×10e7	WT Col-0	non	pathogen
GSE5520_6	WT Col-0	DC3000 hrpA-fliC 10e8	WT Col-0	non	pathogen
GSE5520_7	WT Col-0	DC3000 hrpA 10e8	WT Col-0	non	pathogen
GSE5520_9	WT Col-0	EcO157H7 10e8	WT Col-0	non	pathogen
GSE5526_1	WT?	non	WT?	non	other
GSE5759_1	WT Col-0	dark plus lincomycin	WT Col-0	dark	other
GSE5759_2	WT Col-0	red light plus lincomycin	WT Col-0	red light	other
GSE5770_1	WT Col-0	lincomycin	WT Col-0	non	other
GSE5770_2	MU abi4-102	lincomycin	MU abi4-102	non	other
GSE5770_3	MU gun1-1	lincomycin	MU gun1-1	non	other
GSE630_1	WT Col-0	IAA (2h 5 μM)	WT Col-0	EtOH (2h)	IAA
GSE630_10	MU arf2-6	IAA (2h 5 μM)	MU arf2-6	EtOH (2h)	IAA
GSE630_17	MU IAA17-6	EtOH (2 h)	WT Col-0 I	EtOH (2h)	IAA
GSE630_18	MU arx3-1	EtOH (2 h)	WT Col-0 I	EtOH (2h)	IAA
GSE630_19	MU i5i6i19	EtOH (2 h)	WT Col-0 I	EtOH (2h)	IAA
GSE630_2	MU nph4-1	IAA (2h 5 μM)	MU nph4-1	EtOH (2h)	IAA
GSE630_20	MU IAA17-6	IAA (2h 5 μM)	WT Col-0 I	IAA (2h 5μM)	IAA
GSE630_21	MU arx3-1	IAA (2h 5 μM)	WT Col-0 I	IAA (2h 5μM)	IAA
GSE630_22	MU i5i6i19	IAA (2h 5 μM)	WT Col-0 I	IAA (2h 5μM)	IAA
GSE630_24	MU arf2-6	IAA (2h 5 μM)	WT Col-0 A2	IAA (2h 5μM)	IAA
GSE630_3	MU arf19-1	IAA (2h 5 μM)	MU arf19-1	EtOH (2h)	IAA
GSE630_6	MU IAA17-6	IAA (2h 5 μM)	MU IAA17-6	EtOH (2h)	IAA
GSE630_8	MU i5i6i19	IAA (2h 5 μM)	MU i5i6i19	EtOH (2h)	IAA
GSE631_2	MU arf2-6	IAA (2h 5 μM)	MU arf2-6	non	IAA
GSE631_4	MU arf2-6	IAA (2h 5 μM)	WT Col-0	IAA (2h 5μM)	IAA
GSE911_4	35S::LFY	non	WT ler	35S::LFY	other

Each contrast consists of two groups which are described by their genetic background and treatment. The last column cluster derives from the clustering on the kernel PCA scores. Contrasts are labeled with the GEO series number followed by a contrast index.

## Footnotes

1 http://www.affymetrix.com/products/arrays/specific/arab.affx
